# Defining heatwave thresholds using an inductive machine learning approach

**DOI:** 10.1371/journal.pone.0206872

**Published:** 2018-11-07

**Authors:** Juhyeon Park, Jeongseob Kim

**Affiliations:** School of Urban and Environmental Engineering, Ulsan National Institute of Science and Technology, Ulsan, South Korea; University of California San Francisco, UNITED STATES

## Abstract

Establishing appropriate heatwave thresholds is important in reducing adverse human health consequences as it enables a more effective heatwave warning system and response plan. This paper defined such thresholds by focusing on the non-linear relationship between heatwave outcomes and meteorological variables as part of an inductive approach. Daily data on emergency department visitors who were diagnosed with heat illnesses and information on 19 meteorological variables were obtained for the years 2011 to 2016 from relevant government agencies. A Multivariate Adaptive Regression Splines (MARS) analysis was performed to explore points (referred to as “knots”) where the behaviour of the variables rapidly changed. For all emergency department visitors, two thresholds (a maximum daily temperature ≥ 32.58°C for 2 consecutive days and a heat index ≥ 79.64) were selected based on the dramatic rise of morbidity at these points. Nonetheless, visitors, who included children and outside workers diagnosed in the early summer season, were reported as being sensitive to heatwaves at lower thresholds. The average daytime temperature (from noon to 6 PM) was determined to represent an alternative threshold for heatwaves. The findings have implications for exploring complex heatwave-morbidity relationships and for developing appropriate intervention strategies to prevent and mitigate the health impact of heatwaves.

## Introduction

An extended period of abnormally hot weather (commonly referred to as a heatwave) can cause adverse human health effects. As the frequency, duration and intensity of extreme heat events are predicted to increase due to climate change [1], many countries have implemented heatwave warning systems and response plans to reduce the human health consequences.

Defining a “heatwave” is one key factor in effectively mitigating the impacts of extreme heat events. Certain meteorological thresholds (i.e., two or more consecutive days at a maximum temperature above a certain value) are used for evaluating heatwave extremes and triggering warning systems [2, 3]. Some action plans to protect vulnerable groups have also been designed based on such thresholds.

To establish appropriate heatwave thresholds, many studies have focused on statistically significant increases in relative risk and/or odds ratios under different definitions related primarily to intensity (i.e., maximum daily temperature) and/or duration (i.e., how many days exceed a certain temperature) of temperature [4–12]. A new heat index combining temperature and humidity with apparent temperature is also under consideration to replace existing definitions by comparing odds ratios with heatwave outcomes [13–15]. Previous approaches use deductive reasoning, making assumptions first and then seeking validation using heatwave outcomes.

This study focuses inductively on the non-linear relationship between meteorological variables and heatwave outcomes such as those that take a J, U, or V-shape [16–18]. While a few studies have given attention to the non-linear curve [19, 20], none have investigated a tipping point where human health effects rapidly change, which should be closely related to the definition of a heatwave. This concept has been neglected mainly because classical statistical techniques such as Ordinary Least Square (OLS) methods cannot capture such the tipping point because the linearity and normality assumptions may not be satisfied in the model.

In this paper, we suggest an alternative approach to directly reveal thresholds using Multivariate Adaptive Regression Splines (MARS). This machine learning technique is suitable for capturing curves in predicted outcomes to allow for non-linearity. This approach has been used to develop the heatwave definition by focusing on the relationship between 1) the frequency of emergency department visits identified as heat related and 2) several meteorological factors.

## Materials and methods

### Study area and dataset

**Fig 1:**
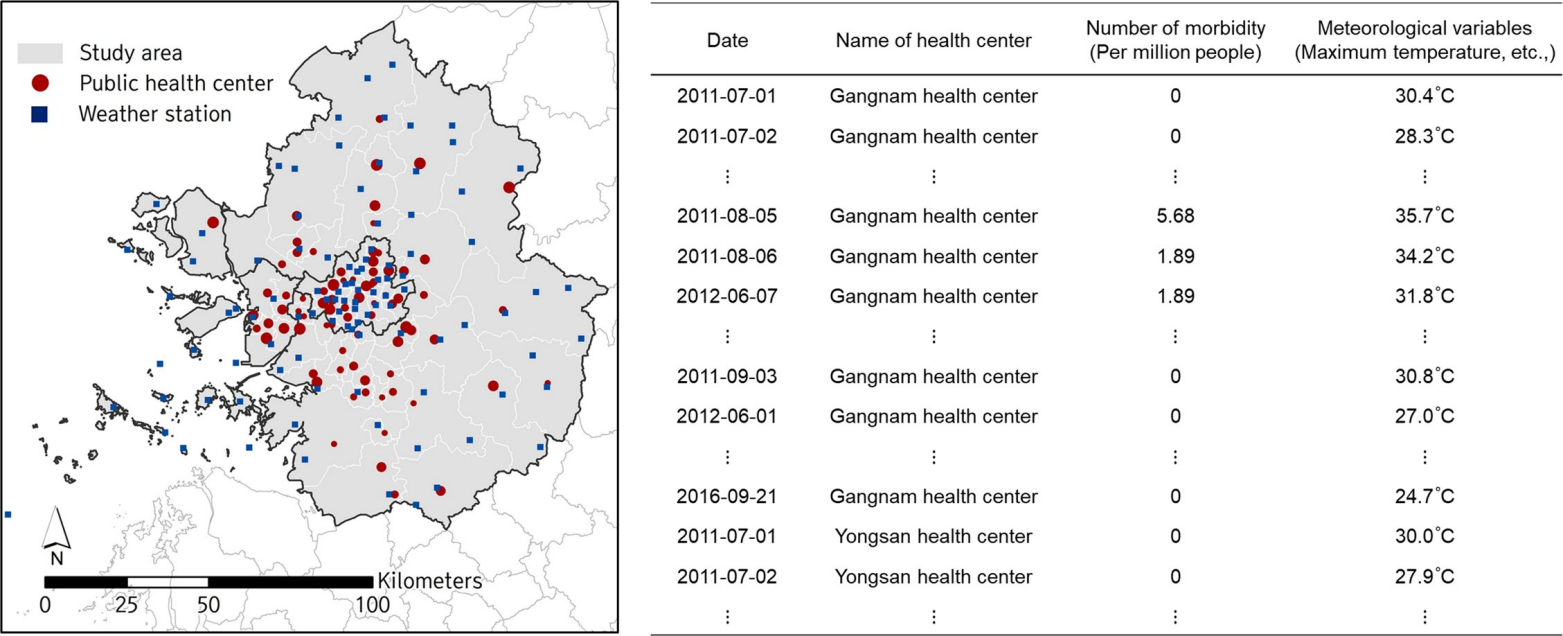
Study area and dataset [Data Source: [21–23]].

We focused on the summer season in the Seoul metropolitan area of Korea, which includes Seoul, Incheon, and Gyeonggi-do, with a population of 25 million in 2015, representing 49.5% of the country’s population. As the Korean Peninsula lies within the East Asian monsoon belt, summer generally falls between June and August, with the hottest month being August when the mean temperature is about 24–26°C. Our dataset included summertime heat-related morbidity rates from 2011–2016 (totaling 521 days from June to August), recorded at 74 public health centers across the region. In other words, there were 38,554 observations considered (74 centers * 521 days). Daily meteorological factors were obtained from the nearest weather station (determined according to the latitudie and longitude of each public health center and each weather station) (Fig 1). The 105 weather stations covered the whole Seoul metropolitan area with an average inter-station distance of 8.7 km and an average of 3.26 km (SD = 2.51 km) from public health centers.

### Data

Daily data on emergency department visitors diagnosed with heat illnesses was obtained from a heat-related illness surveillance system operated by the Korea Centers for Disease Control and Prevention (KCDC) for a total of 586 days during the summer periods from 2011 to 2016: 65 days in 2011 (7/1-9/9), 98 days in 2012 (6/1-9/6), 98 days in 2013 (6/1-9/7), 98 days in 2014 (6/1-9/6), 105 days in 2015 (5/24-9/5) and 122 days in 2016 (5/23-9/21). All cases were initially reported by local emergency medical centers and then collected by the 74 public health centers. The Heat-related illnesses are defined according to the International Classification of Diseases, 10th Revision, T67, “Effects of Heat and Light,” which includes such categories as heat stroke, heat cramps, heat syncope, and heat exhaustion. We extracted the data from June to August from the remaining days because there was a significant amount of missing information for visitors who arrived during the early (May) and late stages (September) of surveillance.

The data included date of visit, sex, age, place of occurence and diagnosis results. This information was categorized by month, place of occurrence, and heatwave vulnerability profile of visitor (age, gender and diagnosis results) (Table 1). To standardize the variables, we divided by the population of the county where each public health center was located (mean = 303,011, SD = 143,257 for the whole population). The population was calculated using the average of 2010 and 2015 census data were obtained from Statistics Korea.

**Table 1:** Description of heatwave morbidity.

(Subcategory) Name		Description	Count (%)
*HWwhole*		Number of heat-related emergency department visits each day	1,468 (100.0)
Age	*HWyoung*	*HWwhole* under 18 years old	87 (5.9)
	*HWadult*	*HWwhole* 18 to 65 years old	1,014 (69.1)
	*HWolder*	*HWwhole* over 65 years old	367 (25.0)
Gender	*HWmale*	Male *HWwhole*	1,161 (79.1)
	*HWfemale*	Female *HWwhole*	307 (20.9)
Diagnosis*	*HWdisch*	*HWwhole* discharged from a hospital	489 (33.3)
	*HWhospital*	*HWwhole* entered a hospital	875 (59.6)
Month of occurrence*	*HWjunjul*	*HWwhole* occurred in June or July	551 (37.5)
	*HWaug*	*HWwhole* occurred in August	917 (62.5)
Place of occurrence*	*HWin*	*HWwhole* occurred indoors	344 (23.4)
	*HWout*	*HWwhole* occurred outdoors	1,102 (75.1)

Note: The variables divided by the population of the county where each public health center was located.

*There is missing information of diagnosis, month and place of occurrence.

Meteorological data (considering 19 factors) from the same time periods was obtained from the Korea Meteorological Administration (Table 2). We adopted the common definition of a heatwave as being a heat event during which a certain temperature threshold is surpassed on a given day (*Tmin*, *Tavg*, and *Tmax*) or over consecutive days (*AvgTmaxLag1*, *AvgTmaxLag2*, and *AvgTmaxLag3*) [24, 25]. Daytime temperature (from noon to 6 PM) and its lagged value were included as alternative heatwave thresholds (*Tavg1218* along with *AvgTavg1218Lag1*, *AvgTavg1218Lag2*, and *AvgTavg1218Lag3*).

**Table 2:** Description of meteorological factors.

Variable	Description	Mean ±std. dev.	Min	Max
*Tmin*_*i*_	Minimum temperature of day i	21.13±2.96	5.00	29.80
*Tavg*_*i*_	Average temperature of day i	24.83±2.51	14.40	32.90
*Tavg1218*_*i*_	Average daytime (noon to 6 PM) temperature of day i	27.68±2.99	13.77	36.44
*AvgTavg1218Lag1*_*i*_	Average *Tavg1218* of day i-1 to i	27.67±2.65	16.22	36.22
*AvgTavg1218Lag2*_*i*_	Average *Tavg1218* of day i-2 to i	27.67±2.47	17.51	35.95
*AvgTavg1218Lag3*_*i*_	Average *Tavg1218* of day i-3 to i	27.66±2.36	17.34	35.76
*Tmax*_*i*_	Maximum temperature of day i	29.5±3.03	16.30	38.90
*AvgTmaxLag1*_*i*_	Average *Tmax* of day i-1 to i	29.5±2.7	18.15	38.80
*AvgTmaxLag2*_*i*_	Average *Tmax* of day i-2 to i	29.49±2.52	19.33	38.47
*AvgTmaxLag3*_*i*_	Average *Tmax* of day i-3 to i	29.48±2.41	19.25	38.13
*TmaxGap*_*i*_	Tmax_i_—Tmax_i-1_	0.42±0.21	0.00	4.10
*TavgGap*_*i*_	Tavg_i_—Tavg_i-1_	0.19±0.07	-0.10	0.55
*Whum*_*i*_	Relative humidity of day i	75.23±13.07	28.90	100.00
*Wpc*_*i*_	Precipitation of day i	8.09±23.38	0.00	449.50
*Wwind*_*i*_	Average wind speed of day i	1.57±0.84	0.00	38.70
*WsolMAX*_*i*_	Maximum amount of solar radiation of day i	2.09±0.82	0.00	3.98
*WsolVOL*_*i*_	Total amount of solar radiation of day i	14.26±6.63	0.00	28.94
*Nindex1*_*i*_	Heat index of day i [26]	74.1±3.9	57.94	85.10
*Nindex2*_*i*_	Discomfort index of day i [27]	79.1±6.86	56.86	107.88

We employed two indices, estimated using combined daily mean temperature and relative humidity to enable a focus on methodological variables, which are simple and not combined. The first index was suggested by the US National Weather Service (NWS), originally developed by Steadman [26] as shown in Eq 1:

$$NIndex1=-42.379+2.04901523*T+10.14333127*RH-0.22475541*T*RH-0.00683783*T^{2}-0.05481717*RH^{2}-0.00122874*T^{2}*RH+0.00085282*T*RH^{2}-0.00000199*T^{2}*RH^{2}$$ (1)

where Nindex1 is the heat index in °F, T is the temperature in °F and RH is relative humidity. Two adjustments were considered when calculating Nindex1 (https://www.wpc.ncep.noaa.gov/heat_index/details_hi.html). Second, a discomfort index (Nindex2) was also established, calculated using the formula by Thom [27] as follows:

$$NIndex2=1.8*T_{c}-(1-0.01RH)(T_{c}-14.3)+32$$ (2)

where T_c_ is the temperature in °C and RH is relative humidity. The maximum value from hourly measurements of the indices was also considered but not adopted because there was multicollinearity with the maximum temperature variables.

### Modelling technique

The MARS method implemented in the “Earth” package for R 3.4.1 was used to investigate the non-linear relationship between heatwave outcomes and meteorological variables. MARS is a spline regression model introduced in 1991 [28] to focus on specific sub-regions of a relationship between covariates and response variables. A knot point *t*, where the behavior of the function changes, marks the end of one region and the beginning of another, forming basis functions: (x-t)_+_ and (t-x)_+_. Firstly, MARS generates a model with an excessive number of knots. Those that contribute least to the overall fit by forward and backward selection are eliminated. A basis function is used to search for the number of knots and their locations, representing the relationships between predictor variables (x) and the outcome variable (y):

$$y=\hat{f}(x)=\beta_{0}+\sum_{i=1}^{k}\beta_{i}h_{i}(x)$$ (3)

where *β*_0_ is an intercpet, *β*_*i*_ is the coefficient estimated by minimizing the sum-of-squares, and *h*_*i*_(*x*) is a weighted sum of basic functions. Initially, The MARS searches all possible basis functions and their corresponding knots using a forward algorithm. Starting with a model consisting of intercept terms, *β*_0_, a larger number of basis functions are added, reducing sum-of-squares residual error as much as possible. However, MARS can be overfit due to a large number of basic functions. To mitigate this problem, a backward phase improves the model by iteratively deleting less significant terms until a final version is reached with the lowest generalized cross validation (GCV). In the model building process, predictors and knot locations that contribute significantly are automatically selected. Additionally, the response variable (y) is defined as a continuous variable because count data cannot be a response variable for the MARS [29, 30].

## Results

Initially, we focused on developing a MARS model based on common meteorological factors (not including *Tavg1218* variables) to explore the definition of a heatwave. The mathematical equation resulting from the MARS model for all emergency department visitors diagnosed with heat illnesses can be expressed as

HWwhole=0.08−0.009*max(0,32.95−AvgTmaxLag1)+0.179*max(AvgTmaxLag1−32.95,0)+0.019*max(Nindex1−79.65,0) (4)

Among all the meteorological variables, *AvgTmaxLag1* and *Nindex1* were included in the equation and others were removed to refine the model fitting process. The knots for *AvgTmaxLag1* and *Nindex1* were 32.95 and 79.65, respectively, and the term “max” can be defined thus: max(j, k) is equal to j if j is larger than k, otherwise it equals k. A positive sign for a function indicates that the relevant meteorological variable increases the probability of a paitient being diagnosed with heat illiness, while a negative sign indicates the relevant variable decreases this probability.

**Fig 2:**
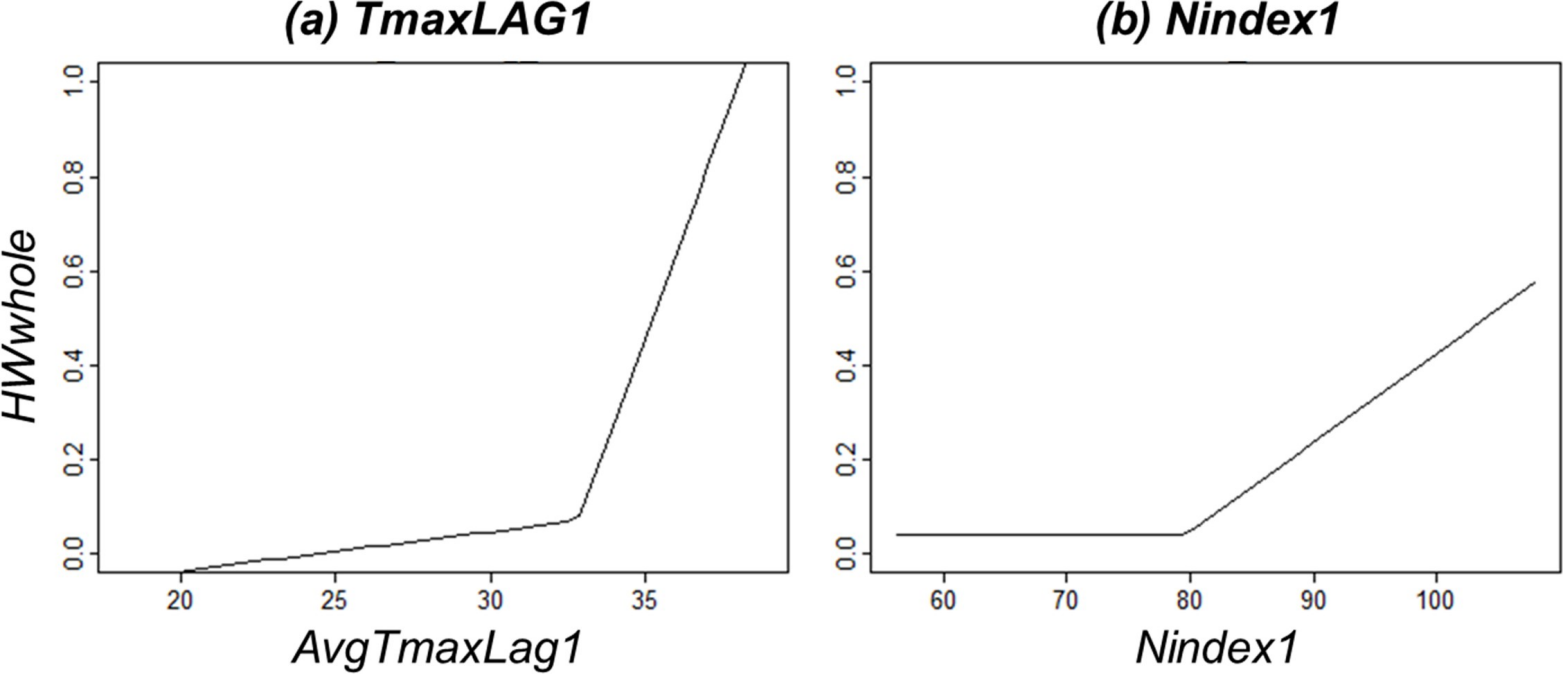
Graphical representation of the MARS model.

Eq 4 can be explained as follows: *AvgTmaxLag1* has little impact on *HWwhole* when *AvgTmaxLag1* is lower than 32.95, while its effect rapidly increases from 32.95. *Nindex1* also influences *HWwhole* after its value becomes larger than 79.65. Fig 2 is a plot of the predicted *HWwhole* as *AvgTmaxLag1* and *Nindex1* vary.

**Fig 3:**
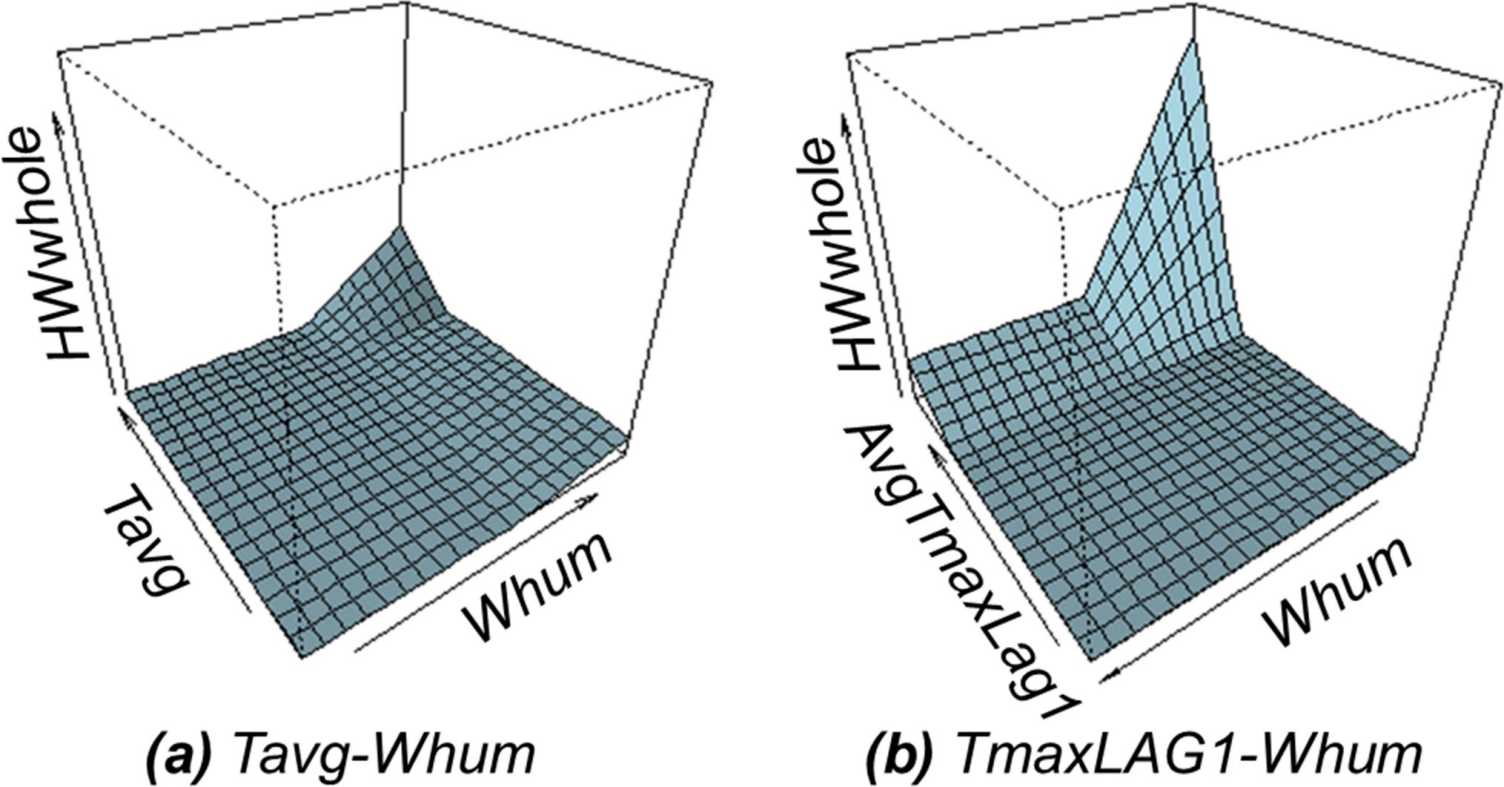
Graphical representation of interaction terms from the MARS.

Moreover, we allowed a second-order interaction term, excluding two combined indexes (*Nindex1* and *Nindex2*). Eq 5 can be formulated, which includes the combination of i) *Tavg-Whum* and ii) *AvgTmaxLag1-Whum* (Fig 3). It denoted that the impact of the temeprature is emerged through the interaction with the humidity in a day.

HWwhole=0.028+0.051*max(Tavg−25.1,0)+1.157*max(AvgTmaxLag1−32.95,0)+0.010*max(Tavg−25.1,0)*max(Whum−81.3,0)+0.030*max(AvgTmaxLag1−32.85,0)*max(0,57.5−Whum−81.3) (5)

Table 3 shows the first and second most important factors, with knots from other MARS models for each subcategorized heatwave outcome and daytime temperature variables. To focus on identifying the locations of knots where the function value was found to vary, we reported each knot and skipped coefficient values. The interpretation for other models was similar to the prior MARS model, which targeted all emergency department visitors diagnosed with heat illnesses, because we verified that the coefficients of all functions in the form “max(x-t, 0)” were positive, as for *AvgTmaxLag1* and *Nindex1* in Eq 4. For example, there is a knot “30.68” of *AvgTmaxLag3* in the model for young heat-related visitors (*HWyoung*). It can be said that the effect of *AvgTmaxLag3* played a role in increasing the frequency of visits by young people when it was larger than the knot (30.68).

**Table 3:** Importance of the MARS model and related knots.

(Subcategory) Outcome		Importance #1		Importance #2	
		Variable	Knot	Variable	Knot
*(Not including Tavg1218 variables)*					
*HWwhole*		*AvgTmaxLag1*	32.95	*Nindex1*	79.65
*Age*	*HWyoung*	*AvgTmaxLag3*	30.68	*-*	-
	*HWadult*	*AvgTmaxLag1*	31.80	*Nindex2*	80.48
	*HWolder*	*AvgTmaxLag1*	33.40	*-*	-
*Gender*	*HWmale*	*AvgTmaxLag1*	32.15	*Nindex1*	78.43
	*HWfemale*	*AvgTmaxLag1*	33.10	*Tavg*	30.5
*Diagnosis*	*HWnothosp*	*AvgTmaxLag1*	32.80	*Nindex2*	76.75
	*HWhosp*	*AvgTmaxLag1*	33.45	*Nindex2*	79.42
*Month of occurrence*	*HWjunjul*	*Tmax*	29.80	*Nindex2*	80.65
	*HWaug*	*AvgTmaxLag1*	33.35	*Nindex1*	
*Place of occurrence*	*HWin*	*Tavg*	30.00	*Nindex2*	78.40
	*HWout*	*AvgTmaxLag1*	31.25	*Nindex2*	80.66
*(Including Tavg1218 variables)*					
*HWwhole*		*AvgTavg1218Lag1*	30.59	*Nindex2*	80.22

## Discussion

This study developed a definition for the heatwave using a machine learning technique, MARS, to describe the fundamental relationship between i) the daily frequency of emergency department visits associated with heat illness, and ii) 19 meteorological factors. MARS enabled non-linear relationships to be rendered and automatically defined breaking points (knots) among separate sub-groups. Knots where the behaviour of functions dramatically changed were used to define a heatwave.

For all emergency department visitors diagnosed with heat illnesses, the related average maximum temperature for 2 consecutive days was greater than 32.58°C and a heat index higher than 79.64 was selected as a threshold based on knots where heatwave morbidity started to rise dramatically. This approach to defining thresholds is similar to existing methods as follows: 1) the Korea Meteorological Administration issues a heatwave warning when the maximum temperature exceeds 33°C for two straight days, while 2) the National Weather Service (NWS) gives a “Caution” notice for possible fatigue from prolonged exposure and/or physical activity when the heat index exceeds 80.

We calculated the number of summer days that meet the existing and alternative criteria based on Station 108, which is representative of Seoul (Table 4). Compared to the current criteria, the number of heat wave days increased three to four times when the two criteria in this study were adopted. When we allowed second-order interactions, combinations of temperature and humidity were included in the model as important variables, even though South Korea only uses a maximum temperature for initiating a heat warning. It suggests that revised criterions and/or more procdure (i.e., attetion, alarm, emegerncy) should be considered for the early warning system.

**Table 4:** Number of summer days that meet existing and alternative criteria based on Station 108 in Seoul.

Year	Number of summer days^HYPERLINK^ ^a^	Existing criteria^b^	Alternative criteria 1^c^	Alternative criteria 2^d^	Alternative criteria 3^e^
2011	92	0	5	27	28
2012	92	10	13	46	46
2013	92	0	4	50	51
2014	92	4	6	37	37
2015	92	3	7	40	41
2016	92	19	29	47	47
Total	552	36	64	247	250

^a^June to October

^b^Maximum temperature exceeds 33°C for two consecutive days

^c^Average maximum temperature for two straight days (AvgTmaxLag1) ≥ 32.58

^d^NWS heat index (Nindex) ≥ 79.64

^e^AvgTmaxLag1 ≥ 32.58 OR Nindex ≥ 79.64

Heat-related outcomes varied depending on visitors’ profiles, similar to findings from previous studies on heat-related mortality and morbidity. Children were reported as being more sensitive to heatwaves [31, 32] as the number of emergency room visitors under 18 years of age entering due to heat illiness was dramatically higher than for other age brackets, even at a lower threshold. While the elderly are also generally considered vulnerable to heatwaves [4, 33–35], an opposite pattern was observed (relatively fewer emergency room visitors even at a higher threshold). As two-thirds of deaths from heatwaves are indeed among the elderly, heat-related mortality was not included in this model: a factor which will be included in future works where more in-depth consideration can be completed. In gender-specific results, males were found to be more likely to visit the emergency room due to heat-related morbidity at a lower threshold compared with females. At a glance, this does not seem consistent with previous studies, which have identified women as facing higher risk or shown no difference by gender [36–39]. The results of this study might explained by males having greater exposure to heat through male-dominated occupations that place individuals in positions vulnerable to heat waves, for example, as construction workers. For diagnosis, it could be helpful to design multi-stage heatwave warning systems according to the threshold for each group, giving sensitivity to differences in demographic occurrences of severe illness and hospital admission.

Our results suggest that heatwave prevention systems and response plans should be designed according to time and place. There is a difference of about 2–3°C in the trigger point for early summer (June to July) for outside workers, a conclusion supported in previous work [40, 41]. In other words, a lower threshold should be set to improve response plans for specific demographics, such that outside workers should take a 10-minute break every hour, as the Korea Occupational Safety and Health Agency recommends.

Additionally, in contrast to the basis for existing thresholds (i.e., daily maximum temperature), this study discovered that average daytime (noon to 6 PM) temperature was determined to represent an alternative threshold for heatwaves as reflecting heat exposures when it is hot during the day. With this in mind, other meteorological factors should be explored as other heatwave thresholds.

Some limitations were encountered during this study. Relying on fixed monitoring stations may misrepresent true individual-level exposures, so additional analyses such as an object analysis of monitoring should be conducted for the meteorological variables considered here. Other heatwave outcomes such as heat-related mortality should also be considered.

## Conclusions

This study investigated heatwave thresholds based on daily data for heat-related morbidity and meteorological variables. Thresholds were inductively determined using MARS models to explore non-linear relationships. For all emergency department visitors diagnosed with heat illnesses, the thresholds identified in this research were similar to existing values used to trigger heatwave warming systems. These thresholds varied depending on visitors’ profiles and the place and time of each occurrence. Average daytime temerpature was selected as an alternative factor informing heatwave thresholds. Our findings can help improve understanding of the effect of heatwaves on human health and be used to design more effective heatwave warning systems.
